# Plant miRNA osa-miR172d-5p suppressed lung fibrosis by targeting Tab1

**DOI:** 10.1038/s41598-023-29188-6

**Published:** 2023-02-06

**Authors:** Motofumi Kumazoe, Fumiyo Ogawa, Ai Hikida, Yu Shimada, Ren Yoshitomi, Ryoya Watanabe, Hiroaki Onda, Yoshinori Fujimura, Hirofumi Tachibana

**Affiliations:** Division of Applied Biological Chemistry, Department of Bioscience and Biotechnology, Faculty of Agriculture, Kyushu University, 744 Motooka, Nishi-Ku, Fukuoka, 819-0395 Japan

**Keywords:** Biotechnology, Molecular biology, Diseases, Health care, Molecular medicine

## Abstract

Lung fibrosis, including idiopathic pulmonary fibrosis, is an intractable disease accompanied by an irreversible dysfunction in the respiratory system. Its pathogenesis involves the transforming growth factorβ (TGFβ)-induced overproduction of the extracellular matrix from fibroblasts; however, limited countermeasures have been established. In this study, we identified osa-miR172d-5p, a plant-derived microRNA (miR), as a potent anti-fibrotic miR. In silico analysis followed by an in vitro assay based on human lung fibroblasts demonstrated that osa-miR172d-5p suppressed the gene expression of TGF-β activated kinase 1 (MAP3K7) binding protein 1 (Tab1). It also suppressed the TGFβ-induced fibrotic gene expression in human lung fibroblasts. To assess the anti-fibrotic effect of osa-miR172d-5p, we established bleomycin-induced lung fibrosis models to demonstrate that osa-miR172d-5p ameliorated lung fibrosis. Moreover, it suppressed Tab1 expression in the lung tissues of bleomycin-treated mice. In conclusion, osa-miR172d-5p could be a potent candidate for the treatment of lung fibrosis, including idiopathic pulmonary fibrosis.

## Introduction

Lung fibrosis is a serious problem affecting the respiratory system^1^. Interstitial pneumonia is caused by several factors, including radiation^2^ and chemotherapeutic drugs, such as bleomycin^3^ and Coronavirus disease 2019 (COVID-19)^4^. Interstitial pneumonia is also a critical problem in cancer immunotherapy^5^. For instance, interstitial pneumonia is a serious problem in immune-related adverse events^5^. Several clinical studies have indicated that immune checkpoint inhibitors, including anti-programmed cell death-1 inhibitors, sometimes cause interstitial pneumonia; once interstitial pneumonia occurs, therapeutic options become limited^6^.

Lung fibrosis, including idiopathic pulmonary fibrosis, is treated with nintedanib and pirfenidone^7^. Although these drugs have significantly enhanced the condition of patients with lung fibrosis in clinical trials^8,9^, prognosis remains to be improved^10^. Long-term observation of 263 patients with idiopathic pulmonary fibrosis has shown that their median survival is 1224 days^11^. Hence, a novel approach must be developed.

MicroRNA (miR) is a form of RNA with approximately 20 short noncoding nucleotides. miRs play a crucial role in our body^12,13^. For example, they act as negative regulators of target genes by directly binding to 3ʹ-untranslated region of a target gene and induces the degradation of the target mRNA or inhibits its translation to proteins^14^. They also participate in 30% of biological processes^15^.

Several studies have described the beneficial effects of plant miRs^16^. In comparison with animal miRs, plant miRs have a robust character because the second hydroxyl group of ribose at the 3ʹ end is methylated^17^. In vivo animal models have also demonstrated that plant miRs elicit anticancer^18^ and suppression effect on LDLR^19^. Recently, absorbed plant MIR2911 suppressed SARS-CoV-2^20^.

Lung fibrosis affects several complicated systems, including oxidative stress^21^, inflammatory cytokine release^22^, immune cell recruitment^23^, and fibroblast activation^24^. In these mechanisms, extracellular matrix overproduction plays a pivotal role in lung fibrosis progression^25^. In this molecular mechanism, transforming growth factorβ (TGFβ) activates fibroblasts and triggers pulmonary fibrosis progression^26^.

TGF-β activated kinase 1 (MAP3K7) binding protein 1, also known as TAK1-binding protein 1 (Tab1), is an adaptor protein in TGFβ signaling^27^. Tab1 directly binds to mitogen-activated protein kinase kinase kinase 7 (TAK1) and regulates its activity^28^. Because of the main role of TAK1 in TGFβ-elicited cascade signaling, Tab1 can also be considered an important component of this system. Therefore, Tab1 could be indispensable in the TAK1 system in vivo^29^.

In this study, osa-miR172d-5p from *Oryza sativa* L. was identified as an anti-fibrotic miR through in silico analysis followed by an in vitro assay using human lung fibroblasts. Osa-miR172d-5p suppressed Tab1 expression and TGFβ-induced fibrosis-related signaling in human lung fibroblasts. A bleomycin-induced pulmonary fibrosis model, which is the standard mouse model of lung fibrosis, was developed to demonstrate that osa-miR172d-5p suppressed lung fibrosis and downregulated Tab1 expression. Importantly, this study was the first to describe that Tab1 suppression could ameliorate bleomycin-induced fibrosis. Therefore, osa-miR172d-5p could be a potent candidate for establishing novel strategies of pulmonary fibrosis therapy.

## Results

### Osa-miR172d-5p was identified as a plant miR candidate with anti-fibrotic effects

MiRs are known as negative gene expression regulators that degrade target mRNA or inhibit translation^12–14^. The abnormal activation of TGFβ signaling plays an indispensable role in pulmonary fibrosis^26^, including idiopathic pulmonary fibrosis^30^. In this study, we focused on plant miRs targeting the TGFβ signaling cascade because they are relatively stable due to their unique 3′ modification^18^. Recent reports based on next-generation sequencing have also revealed the presence of plant miRs in human blood^31^.

**Figure 1:**
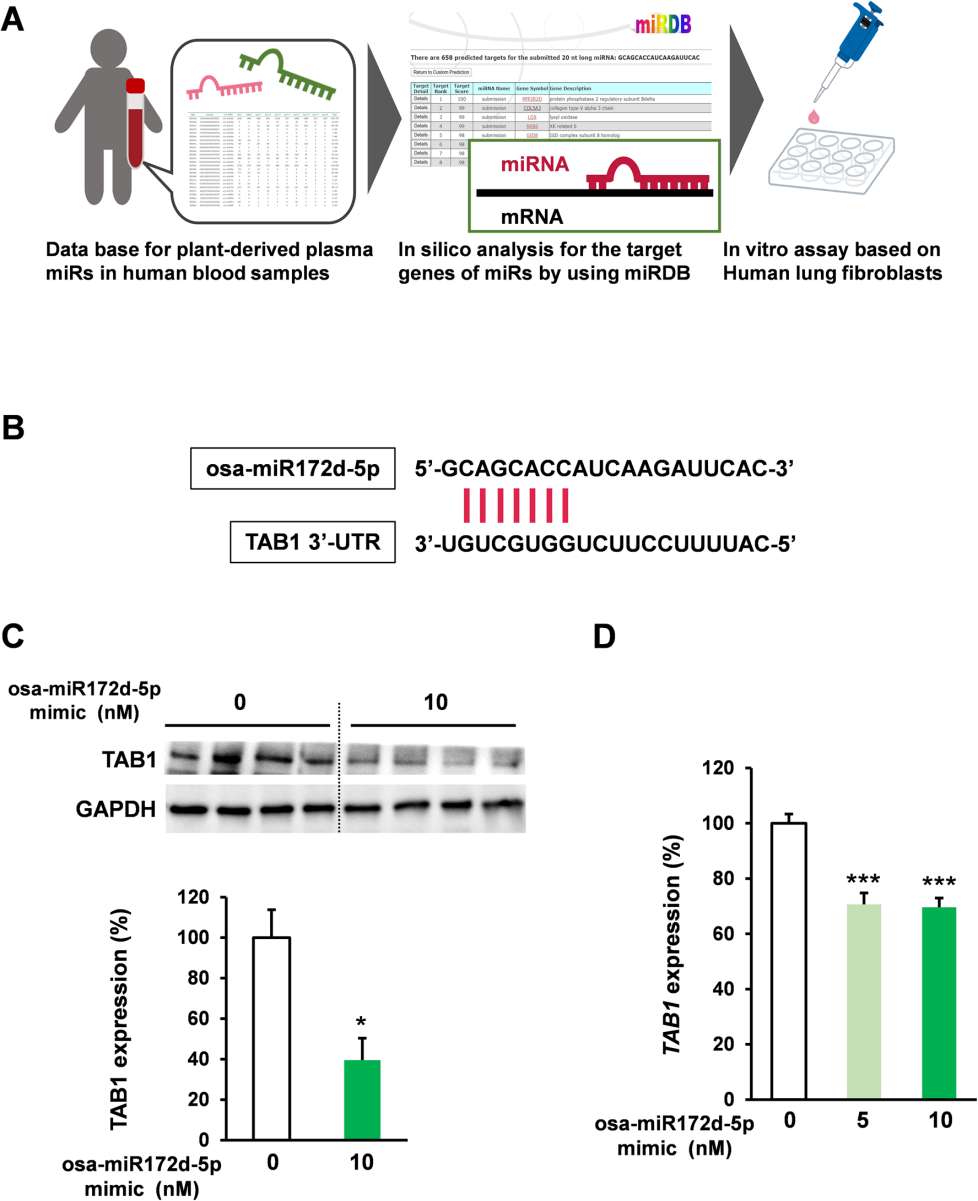
Osa-miR172d-5p identified as a plant miR candidate with an anti-fibrotic effect. (**A**) Scheme of plant miR selection. (**B**) In silico analysis of the interaction between osa-miR172d-5p and TAB1. (**C**) Human lung fibroblast HFL1 cells were transfected with the osa-miR172d-5p for 48 h, and TAB1 expression was evaluated via western blot analysis (*n* = 4). (**D**) HFL1 cells were transfected with the indicated concentration of osa-miR172d-5p for 48 h, and cDNA was evaluated via qRT-PCR (*n* = 4). Data are shown as mean ± SEM. *P < 0.05. ***P < 0.001 versus control group.

Considering stability, we investigated plant miRs that could be detected in human blood have effects on fibroblasts those plays the crucial role in fibrosis^32–34^. On the basis of a previous report on plant miR information and in silico analysis (miRDB, URL) of miR targets for anti-fibrotic effects^35^, osa-miR172d-5p was identified as a plant miR candidate with anti-fibrotic effects (Fig. 1A) by targeting TAB1, which is an important component in TGFb signaling^27^ (Fig. 1B). Human lung fibroblast HFL1 cells were treated with osa-miR172d-5p and the TAB1 protein expression was evaluated via western blot after 48 h to assess the inhibitory effect of osa-miR172d-5p on TAB1 expression. Immunoblot analysis revealed that osa-miR172d-5p suppressed the TAB1 protein expression (Fig. 1C). Furthermore, HFL1 cells were treated with osa-miR172d-5p, and the TAB1 mRNA expression was evaluated through qPCR after 48 h (Fig. 1D) to examine the effect of osa-miR172d-5p on the TAB1 mRNA expression. The results demonstrated that osa-miR172d-5p suppressed the mRNA TAB1 expression. Therefore, osa-miR172d-5p was identified as an anti-fibrotic plant miR candidate.

### Osa-miR172d-5p suppressed TGFβ-induced fibrotic gene expression

**Figure 2:**
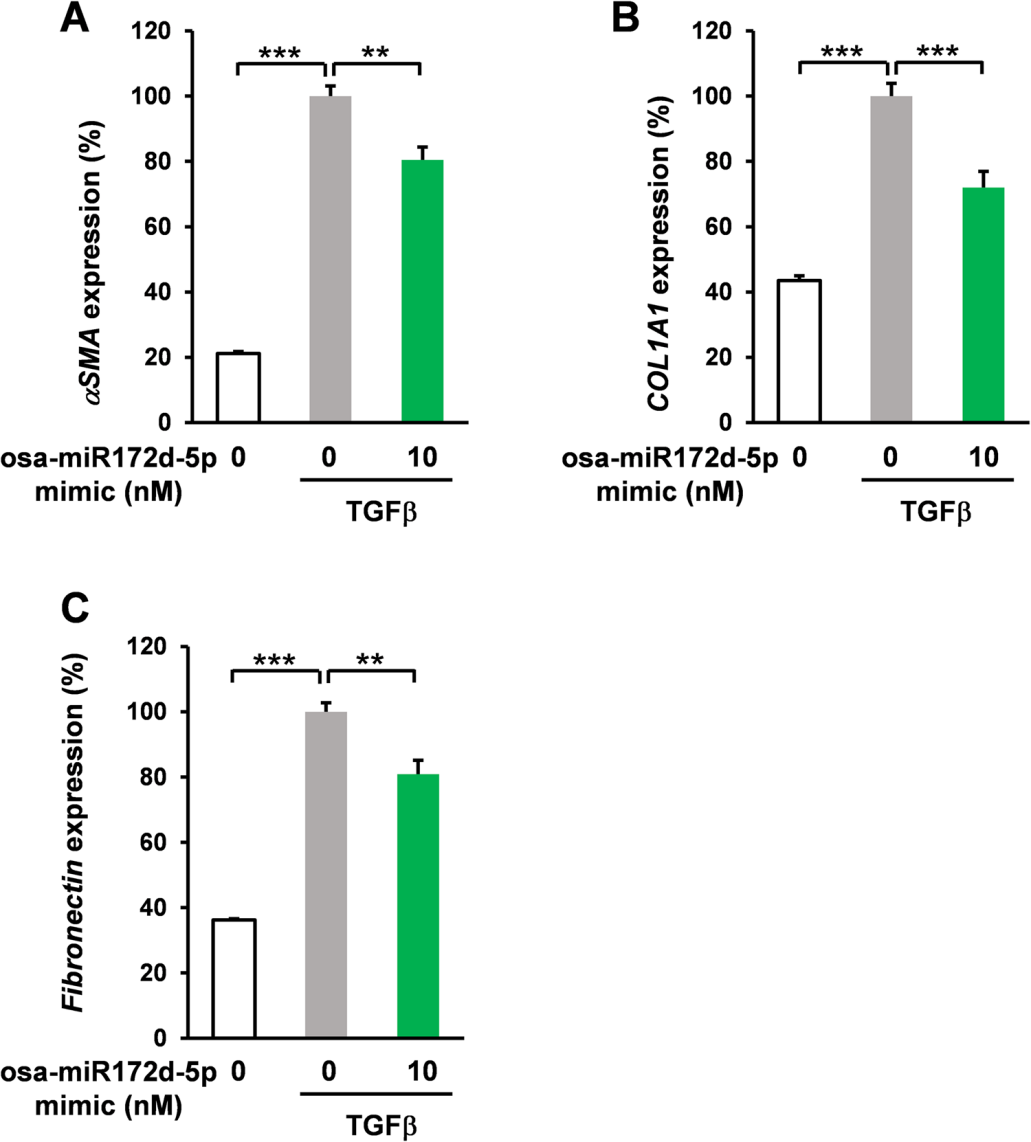
Osa-miR172d-5p suppressed TGFβ-induced fibrotic gene expression. (**A**–**C**) Human lung fibroblast HFL1 cells were transfected with osa-miR172d-5p (10 nM, 48 h) and treated with TGFβ (5 ng/mL for 48 h). mRNA expression levels were assessed via RT-qPCR. (A) ASMA (αSMA) (n = 4), (**B**) COL1A1 (n = 4), and (**C**) FN (fibronectin; n = 4). Data are shown as mean ± SEM. **P < 0.01. ***P < 0.001 versus control group.

Abnormally activated fibroblasts produce collagens and other extracellular matrices^32^. The accumulation of the extracellular matrix in the lungs irreversibly deteriorated the respiratory function by decreasing the diffusing capacity of the lungs and lung compliance^33^. In these molecular mechanisms, TGFβ triggers lung fibroblast activation^26^. In silico analysis and cell-based assay indicated that osa-miR172d-5p suppressed the TAB1 expression, which is a crucial mediator of the TGFβ signaling cascade^27,28^; therefore, the effect of osa-miR172d-5p on TGFβ-induced fibrotic signaling was assessed. HFL1 cells were pretreated with osa-miR172d-5p (10 nM, 48 h) and treated with TGFβ (5 ng/mL, 48 h), and gene expression levels were evaluated via qRT-PCR. Alpha smooth muscle actin (ASMA) is a well-known marker of myofibroblast differentiation^34^. It is highly expressed in fibroblasts from patients with pulmonary fibrosis^35^ (Fig. 2A), and its upregulation was induced by TGFβ, but these effects were impeded by osa-miR172d-5p treatment (Fig. 2A). TGFβ treatment also induced collagen I and fibronectin expression in HFL1 cells (Fig. 2B,C). Conversely, osa-miR172d-5p treatment reduced the extracellular matrix expression (Fig. 2B,C). Therefore, osa-miR172d-5p treatment ameliorated TGFβ-induced fibrosis in lung fibroblasts. We also assessed the effect of osa-miR172d-5p (10 nM, 24 h and 48 h) on the viable cell number of HFL1 cells. Our results demonstrated that osa-miR172d-5p did not affect the viable cell number (SI Fig. 1). Moreover, we assessed the effect of osa-miR172d-5p on the expression levels of senescence related genes (Interleukin-6 (IL6), Glutaminase-1 (GLS), Interleukin-1β (IL1β), cyclin-dependent kinase inhibitor 1 (P21)) and autophagy related genes (Autophagy Related 16 Like 2 (ATG16L2), beclin1 (BECN), and C/EBP homologous protein (CHOP) (SI Fig. 2). Our results indicated that osa-miR172d-5p suppressed the expression of P21 (SI Fig. 2B). GLS1, IL1β, ATG16L2, and BECN gene expression were difficult to detect (more than 30% of samples had undetectable levels). Notably, as P21 is also regulated by TGFβ signaling, this event could be involved in the protective effect of osa-miR172d-5p in the lungs. We also assessed the effect of osa-miR172d-5p (10 nM, 48 h) on human pulmonary artery endothelial cells (HPAECs). We confirmed that there was no effect on the cell growth of HPAECs (SI Fig. 3A). Our results showed osa-miR172d-5p did not affect the ASMA, P21 and GLS1 expression levels in HPAECs (SI Fig. 3B-D). Moreover, IL6, TAB1, and IL1β expression levels were difficult to detect (more than 30% of samples had undetectable levels). These data indicated that osa-miR172d-5p has protective effect on the lung through the fibroblast-dependent mechanism.

**Figure 3:**
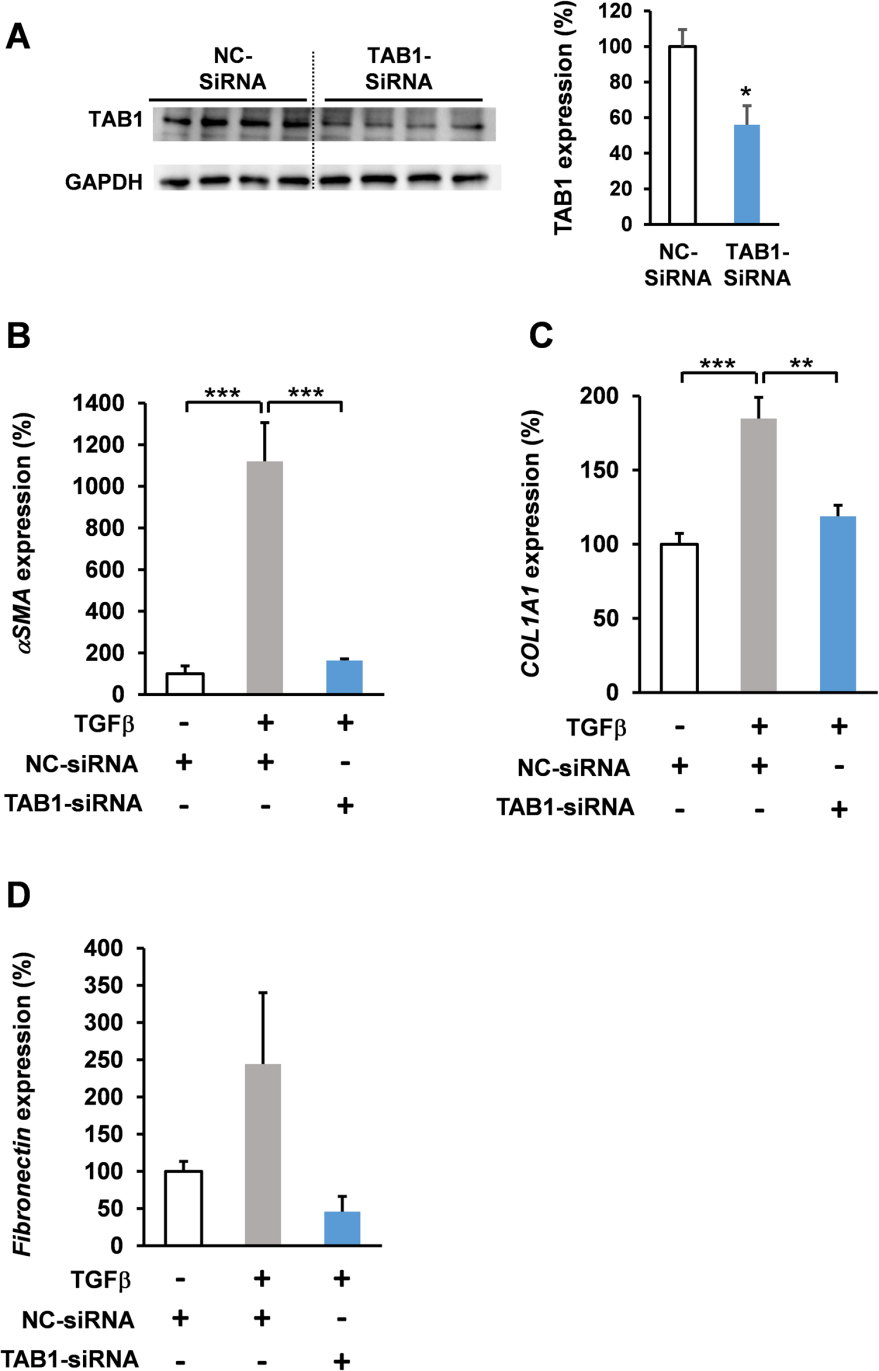
TAB1 knockdown suppressed TGFβ-induced fibrotic gene expression. (**A**) Human lung fibroblast HFL1 cells were transfected with TAB1-siRNA (10 nM, 48 h) and TAB1 expression levels were determined by western blot analysis. (**B**–**D**) Human lung fibroblast HFL1 cells were transfected with TAB1-siRNA (10 nM, 48 h) and treated with TGFβ (5 ng/mL for **B**, **D** 48 h; **C** 24 h). mRNA expression levels were assessed via RT-qPCR. (**B**) ASMA (αSMA) (n = 4), (**C**) COL1A1 (n = 4), and (**D**) FN (fibronectin; n = 4). Data are shown as mean ± SEM. *P < 0.05.  **P < 0.01. ***P < 0.001 versus control group.

To assess the impact of suppressing TAB1 expression on the TGFβ signaling in fibroblasts, HFL1 cells were treated with siTAB1 RNA for 48 h (Fig. 3A–D), and then with TGFβ (5 ng/mL) (Fig. 3B–D). The gene expression levels were determined using qPCR analysis. Our results demonstrated that silencing TAB1 suppressed the TGFβ-induced fibrosis signaling (Fig. 3B,C).

### Osa-miR172d-5p attenuated bleomycin-induced fibrosis in vivo

**Figure 4:**
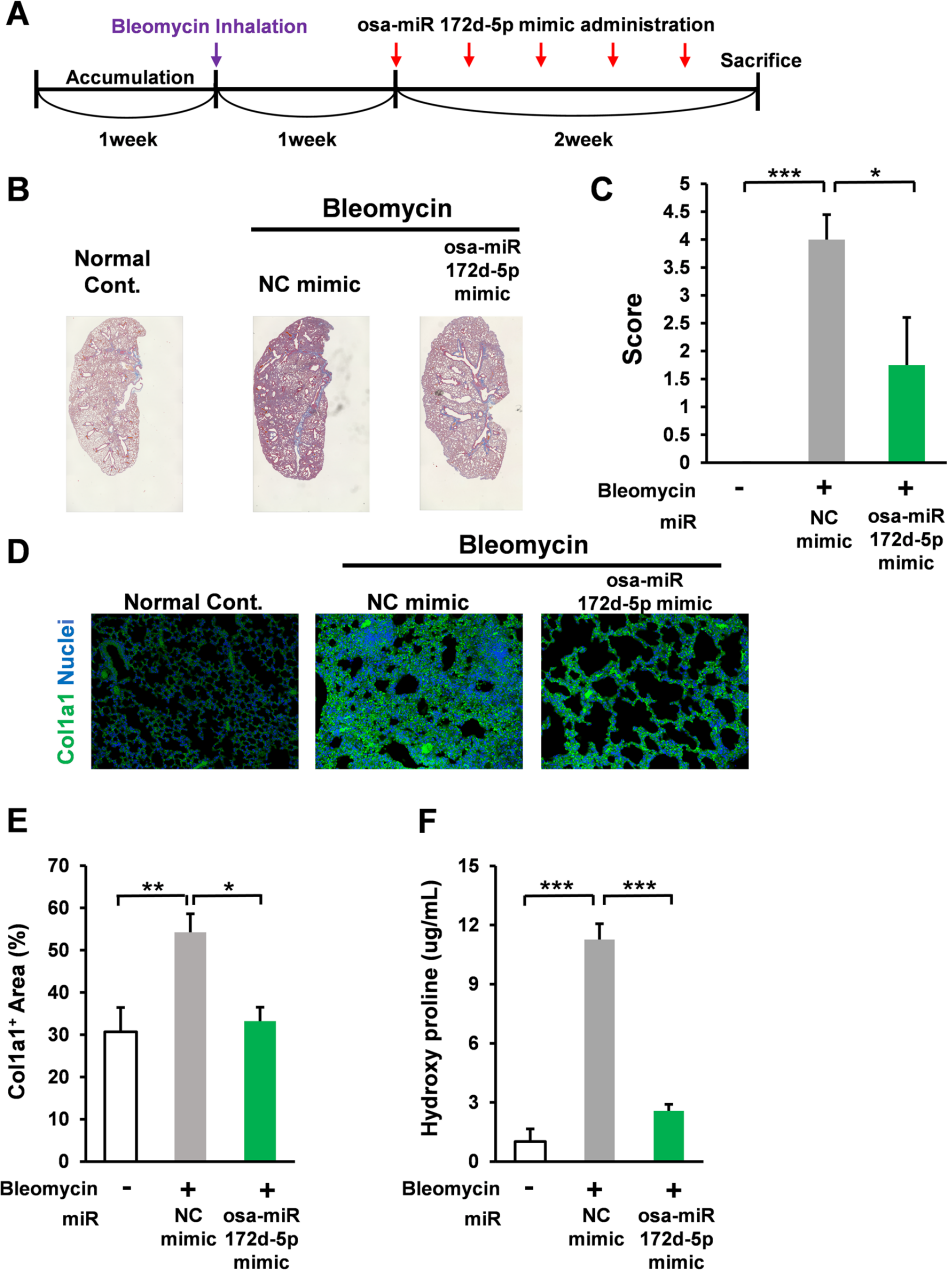
Osa-miR172d-5p attenuated bleomycin-induced fibrosis in vivo. (**A**) Scheme of bleomycin-induced pulmonary fibrosis model. (**B, C**) C57BL male mice were treated with bleomycin (1 mg/kg bw intratracheal administration); after 1 week, the bleomycin treated-mice were treated with the negative control miR (3 nmol/mouse five times in 2 weeks, intraperitoneal injection with AteloGene) or miR Osa-miR172d-5p mimic miR (3 nmol/mouse five times in 2 weeks, intraperitoneal injection with AteloGene). After 3 weeks, the mice were sacrificed, their lung tissues were harvested, and fibrosis was assessed via Masson’s trichrome staining (n = 5, 5, 4). (**D, E**) Immunofluorescence image of Col1a1 in mouse lung tissues (n = 5, 5, 4). (**F**) Western blot analysis of Col1a1 in mouse lung tissues (n = 5, 5, 4). Data are shown as mean ± SEM. *P < 0.05. **P < 0.01 ***P < 0.001 versus control group.

Bleomycin-induced pulmonary fibrosis model is widely used to study lung fibrosis, including idiopathic pulmonary fibrosis^36^. In accordance with the standard model, C57BL male mice were treated with bleomycin (1 mg/kg bw intratracheal administration); after 1 week, the bleomycin-treated mice were administered with the negative control miR (3 nmol/mouse five times in 2 weeks, intraperitoneal injection with AteloGene) or miR osa-miR172d-5p mimic (3 nmol/mouse five times in 2 weeks, intraperitoneal injection with AteloGene). After 3 weeks, the mice were sacrificed, and lung tissues were harvested (Fig. 4A).

Masson’s trichrome staining is mainly performed to evaluate pulmonary fibrosis^37^. In this method, the cytoplasm is stained red, and collagen is stained with aniline blue dye. In this study, Masson’s trichrome staining revealed that bleomycin administration significantly induced fibrosis in the lung tissues of the negative-control mimic-treated mice. However, the fibrotic effect was attenuated by osa-miR172d-5p treatment compared with that by the negative control mimic (Fig. 4B,C).

Collagen accumulation is the characteristic change in idiopathic pulmonary fibrosis^38^. Excessive extracellular matrix accumulation compromised the respiratory function caused by the reduction of lung compliance^38^. Immunofluorescence analysis of the lung section demonstrated that bleomycin treatment induced the collagen 1-positive area, and bleomycin-induced increase in collagen 1 was significantly suppressed by osa-miR172d-5p treatment compared with that by the negative control mimic (Fig. 4D,E). Consistent with IHC analysis, our western blotting analysis also demonstrated the inhibitory effect of osa-miR172d-5p on bleomycin-induced fibrosis (SI Fig. 4A,B)”.

Hydroxyproline is a well-established marker of lung fibrosis. Our results showed that osa-miR172d-5p administration negated bleomycin-induced upregulation of hydroxyproline levels in lung (Fig. 4F). Therefore, osa-miR172d-5p treatment showed an anti-fibrotic effect in vivo.

### Osa-miR172d-5p suppressed Tab1 upregulation in bleomycin-induced fibrosis model

**Figure 5:**
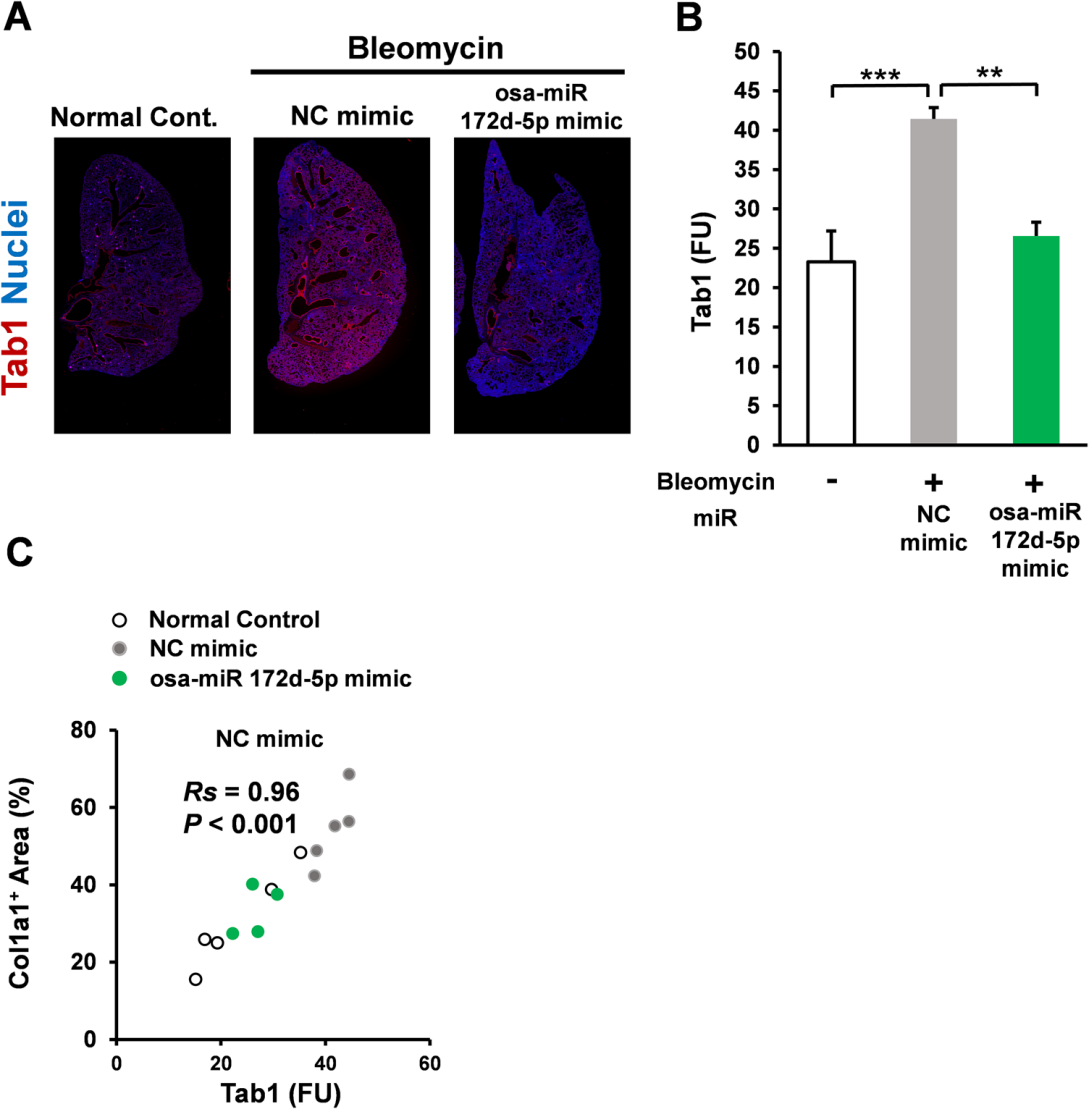
Osa-miR172d-5p suppressed Tab1 upregulation in a bleomycin-induced fibrosis model. (**A, B**) C57BL male mice were treated with bleomycin (1 mg/kg bw intratracheal administration); after 1 week, the bleomycin-treated mice were treated with the negative control miR (3 nmol/mouse 5 times in 2 weeks, intraperitoneal injection with AteloGene) or miR Osa-miR172d-5p mimic miR (3 nmol/mouse five times in 2 weeks, intraperitoneal injection with AteloGene). After 3 weeks, the mice were sacrificed, their lung tissues were harvested, and fibrosis was assessed through the immunofluorescence analysis of Tab1 in mouse lungs (n = 5, 5, 4). (**C**) Correlation was performed via Spearman’s rank test (n = 14). Data are shown as mean ± SEM. **P < 0.01. ***P < 0.001 vs. control group.

Because the in vitro analysis of osa-miR172d-5p indicated TAB1 as its target, the effect of osa-miR172d-5p treatment on Tab1 levels in bleomycin-treated mice was examined. Those data showed that Tab1 expression levels were downregulated in the mice treated with osa-miR172d-5p compared with those in mice treated with the negative control mimic (Fig. 5A,B). The Tab1 expression was significantly correlated with Col1-positive area (Spearman’s rank test, *Rs* = 0.96, *P* < 0.001, n = 14; Fig. 5C). Therefore, plant-derived miR osa-miR172d-5p suppressed Tab1 in the bleomycin-induced fibrosis model.

## Discussion

Although several therapeutic approaches have been developed for pulmonary fibrosis, the prognosis of patients with pulmonary fibrosis remains poor (median: 4 years)^11^. Thus, novel strategies should be developed.

TGFβ signaling is implicated in pulmonary fibrosis progression^26^. In clinical settings, TGFβ signaling overactivation is observed^39^. However, TGFβ is involved in several physiological processes, including cancer prevention^40^, differentiation^41^, and immune tolerance^42^; therefore, targeting TGFβ itself cannot be applied because of adverse effects^43^.

In this study, osa-miR172d-5p suppressed TAB1 expression in human lung fibroblasts and suppressed TGFβ-induced fibrosis-related signaling. Using a bleomycin-induced pulmonary fibrosis model, we demonstrated that osa-miR172d-5p suppressed lung fibrosis and Tab1 expression. We also showed that silencing TAB1 is sufficient to negate the TGFβ-induced fibrosis related gene expression.

Although the essential role of TAB1 in TGFβ signaling has been described^27^, the effect of Tab1 suppression on lung fibrosis has not been determined. Our immunohistochemical analysis showed that Tab1 was upregulated in bleomycin-treated mice compared with their normal counterparts. These findings suggested that Tab1 could be a potent candidate to selectively suppress fibrotic signaling. Considering the inhalation of short miR has been developed^44^, and those would provide advantage for this miR because miRs locally suppress TGFβ signaling in the lungs and consequently evade adverse effects.

In a recent study, it was shown that Tab1 in the epithelial cells plays a crucial role in the fibrosis process^45^. TGFβ signaling in the fibroblast is a major process in the fibrosis and, osa-miR172d-5p suppresses the TGFβ-induced fibroblast signaling; however, Tab1 in the epithelial cells could also be a target of this microRNA.

The dose used in this study (3 nmol/mouse) is the standard in the region. Studies have shown that the oral intake of plant miRs may cause the efflux of miRs into the blood^31^. For instance, some plant miR levels increase in the plasma after the oral administration of watermelon juice (22-year-old male volunteers weighing approximately 60 kg), and the highest *C*_max_ (MIR 528) was 16.14178 pM with an absorption rate of 1.31%^31^. However, further studies should determine the role of plasma osa-miR172d-5p derived from dietary intake.

In pulmonary fibrosis, acute exacerbation is a major problem^46^. Acute exacerbation of idiopathic pulmonary fibrosis is caused by infections, including influenza^46^. Previous retrospective reviews showed that acute exacerbation is caused by infections (55.2%), and its incidence (3 years) is 20.7%. Acute exacerbation is an important predictor of poor survival (1-year survival rate of 56.2%)^46^. Considering that inflammation is crucial in acute exacerbation and that Tab1 is indispensable in inflammatory signaling^47^, osa-miR172d-5p may ameliorate acute exacerbation in idiopathic pulmonary fibrosis.

In conclusion, osa-miR172d-5p suppressed Tab1 overexpression in bleomycin-treated mice, TGFβ-elicited fibrosis signaling in human lung fibroblasts, and bleomycin-induced fibrosis in the lungs. Therefore, osa-miR172d-5p could be a potent candidate for pulmonary fibrosis therapy.

## Materials and methods

### Materials and reagents

Recombinant human TGFβ (100-B-001; R and D Systems, McKinley Place, NE, USA) was dissolved in 0.1% bovine serum albumin (BSA) (A1933, Sigma-Aldrich, St. Louis, MO, USA) with HCl (4 mM) at 10 µg/mL. The following materials and reagents were also used: Lipofectamine™ RNAiMAX transfection reagent (13778150; Invitrogen, Waltham, MA, USA); bleomycin (B3972; Tokyo Chemical Industry Co., Ltd., Tokyo, Japan); mouse anti-col1a antibody (sc-59772; Santa Cruz Biotechnology, Santa Cruz, CA, USA); rabbit anti-Tab1 antibody (ab25878; Abcam, Cambridge, UK); Hoechst 33342 (H3570; Invitrogen); osa-miR172d-5p mimic (Fasmac, Tokyo, Japan); Alexa fluor 488-labeled anti-mouse antibody Fab fragment (A11017) and Alexa fluor 555-labeled anti-rabbit antibody Fab fragment (A21428; Invitrogen); phosphate-buffered saline (PBS) (045-29795; Fuji Firm, Tokyo, Japan); AteloGene (1391; Koken, Tokyo, Japan); 4% paraformaldehyde (163-20145) and Lemosol (128-03993; Fujifilm, Tokyo, Japan); Vectashield (H-1000; Vector Laboratories, Burlingame, CA, USA); and Trans Blot nitrocellulose membranes (Protran BA 85; Sigma Aldrich). miRNA data were obtained from previous reports^19,48,49^ followed by miRDB (http://www.mirdb.org/).

### Cell culture and assay

Human lung fibroblast HFL1 cells (JCRB, Osaka, Japan) were cultured in 10% fetal bovine serum (FBS) (Sigma-Aldrich), Dulbecco's Modified Eagle Medium (DMEM) (044-29765; Fujifilm Tokyo, Japan) supplemented with penicillin G (876111)–streptomycin (876161; Meiji Pharmaceutical Co., Tokyo, Japan) in 5% CO_2_ and 100% humidity at 37 °C. HPAECs (Kurabo, Kurashiki, Japan, KA-4109) were cultured in EnGS complete medium (1% FBS EnGS; VascuLife EnGS LFactors, Kurabo, Kurashiki, Japan, LEK-LS1019) with 5% CO_2_ and 100% humidity at 37 °C. In western blot analysis, HFL1 cells were seeded on a 24-well plate at a density of 1 × 10^5^ cells/mL in 10% FBS-DMEM. After 24 h of preculture, the medium was replaced with 1% FBS-DMEM, and the cells were treated with the indicated concentrations of osa-miR172d-5p by using Lipofectamine™ RNAiMAX transfection reagent in accordance with manufacturer’s protocol. Then, they were cultured for 48 h and harvested with cell lysis buffer. HFL1 cells were inoculated into 96-well plates (1 × 10^5^ cells/mL in 200 μL 10% FBS DMEM). After 24 h, medium was changed (1% FBS DMEM), and treated with indicated miRs for 24 h, 48 h. Cells were assessed using ATPlite 1 step (6016731 ParkinElmer Waltham MA, USA). HPAECs were inoculated into 96-well plates (1 × 10^4^ cells/mL in 200 µL 1% FBS EnGS). After 24 h, medium was changed (1% FBS EnGS 200 µL), and the cells were treated with each concentration of indicated miRs for 48 h. Cells were assessed using ATPlite. HPAECs were inoculated into 12-well plates (1 × 10^5^ cells/mL in 2 mL 1% FBS EnGS). After 24 h, medium was changed (1% FBS EnGS 400 μL), and treatment with indicated miRs was performed for 48 h. HFL1 cells were inoculated into 24-well plates (0.75 × 10^5^ cells/mL in 1 mL DMEM supplemented with 10% FBS). After 24 h, medium was changed (DMEM supplemented with 1% FBS), and treatment with indicated siRNA (SiTab1 Silencer™ Pre-Designed siRNA, ID: 17476) was performed for 48 h. Cells were then treated with TGFb (5 ng/mL) and harvested.

### Western blot

Harvested lysate (approximately 10 μg of protein) was diluted with the sample buffer (1:1; 10% glycerol, 0.001% bromophenol blue, 1% sodium dodecyl sulfate [SDS], and 0.10 M Tris–HCl buffer with 0.05% 2ME; pH 6.8). The mixed lysate solution was heated (at 95 °C heat block for 5 min), electrophoresed via SDS-PAGE (0.03 A, 1.5 h), and transferred to Trans Blot nitrocellulose membranes (100 V, 1 h). The membranes were blocked with a blocking buffer (2.5% BSA Tween 20-TBS [TTBS]) for 1 h, incubated with a primary antibody (1:3000 anti-TAB1 antibody diluted with 2.5% BSA TTBS), and stored at 4 °C overnight. Then, they were washed with TTBS, incubated with secondary antibodies (horseradish peroxidase [HRP]-labeled secondary antibodies at 1:10,000) for 1 h, and washed again with TTBS. All membranes were evaluated with a chemiluminescence solution (Lumigen, ECL Ultra, TMA-6) in the Fusion System (Vilber-Lourmat, Collégien, France) by using Kyplot 6.0 (KyensLab Inc., Tokyo, Japan). Blots were cut prior to hybridisation with antibodies.

### qRT-PCR assay

In the qRT-PCR analysis for miR confirmation, HFL1 cells were seeded on a 24-well plate at a density of 1 × 10^5^ cells/mL in 10% FBS-DMEM. After 24 h of preculture, the medium was replaced with 1% FBS-DMEM, and the cells were treated with the indicated concentrations of osa-miR172d-5p by using Lipofectamine™ RNAiMAX transfection reagent in accordance with the manufacturer’s protocol. Then, they were cultured for 48 h and harvested with 700 μL of Tri-Reagent (Cosmo Bio, Tokyo, Japan). In the qRT-PCR analysis of the anti-fibrotic effect of osa-miR172d-5p, HFL1 cells were seeded on a 24-well plate at a density of 0.75 × 10^5^ cells/mL in 10% FBS-DMEM. After 24 h of preculture, the medium was replaced with 1% FBS-DMEM, and the cells were treated with 10 nM osa-miR172d-5p or scramble miR by using Lipofectamine™ RNAiMAX transfection reagent in accordance with the manufacturer’s protocol. Afterward, they were cultured for 48 h, treated with TGFβ (5 ng/mL for 48 h), and harvested with 700 μL of Tri-Reagent.

Then, 200 μL of CHCl_3_ was added to 700 μL Tri-Reagent. After 3 min at room temperature-incubation, samples were centrifuged (12,000×*g*, 4 °C for 15 min). An equal volume of 2-propanol was added to the collected water layer. After 10 min of incubation at room temperature, the samples were centrifuged (12,000×*g* and 4 °C for 15 min), and the supernatant was removed. Then, 800 μL of 75% EtOH was added, the samples were centrifuged (12,000×*g* and 4 °C for 5 min), and the supernatant was removed. Pellets were dissolved in nuclease-free water (Ambion, MA, USA), and RNA concentrations were determined using NanoDrop 2000 (Thermo Fisher Scientific, MA, USA). Thereafter, 100 ng/mL RNA solution was used for to synthesize the cDNA with a Prime Script RT reagent kit (RR037A, Takara Bio, Siga, Japan) and a thermal cycler (Astec, Fukuoka, Japan) in accordance with the manufacturer’s protocol. cDNA was evaluated via qRT-PCR by using the CFX 96Real-Time PCR System (Bio-Rad, Hercules, CA) with SsoAdvanced Universal SYBR Green Supermix (172-5271, Bio-Rad) in accordance with the manufacturer’s protocol.

### Animal study

Animal experiments were performed in accordance with Notification no. 6 and Regulation no. 105 in Japan and approved by the Animal Care and Use Committee (Kyushu University, Fukuoka, Japan). However, because of the lack of information about the efficacy of osa-miR172d-5p on lung fibrosis, group size could not be calculated. All animals were maintained in a controlled room with approximately 60% humidity at approximately 20 °C in a 12 h dark–light cycle (dark from 08:00 to 20:00). The mice were provided diet (MF diet, KBT oriental Saga, Japan) and drinking water (autoclaved deionized water) ad libitum. This research was reported in accordance with ARRIVE guidelines.

C57BL6J male mice (Kyudo, Tosu, Japan) were randomly grouped into three cohorts: **Gp1** normal group, **Gp2** control group, and **Gp3** miR osa-miR172d-5p.

At 6 weeks of age, Gp1 mice were intratracheally administered with PBS (80 μL/mouse) under isoflurane vapor on day 0. Gp2 and Gp3 mice were intratracheally administered with bleomycin PBS (80 μL/mouse; bleomycin 1 mg/kg b.w.) under isoflurane vapor on day 0.

On days 6, 9, 12, 15, and 19, Gp2 mice were intraperitoneally (IP) injected with scramble miR (3 nmol/mouse in AteloGene), and Gp3 mice were IP injected with osa-miR172d-5p (3 nmol/mouse in AteloGene). All mice were sacrificed on day 21 under isoflurane vapor. The lungs were collected and further analyzed. All alive mice were included and assigned randomly to the groups without blinding. In all animal studies, the unit was the mouse. Hydroxyproline levels were determined by using Hydroxyproline assay kit (QuickZyme Biosciences, Netherlands, Leiden, QZBHYPRO1). Briefly, lung tissue was treated with 6 M HCl (100 mg tissue/mL) at 95 °C for 24 h and samples were centrifuged at 13,000 g for 10 min, and supernatant was harvested. All procedures were performed in accordance with its manufacture’s protocol.

### Histological analysis and immunofluorescence chemistry

Harvested lungs were soaked with 4% paraformaldehyde for 1 week, embedded in paraffin (Kyodo Byori, Kobe, Japan), deparaffinized with Lemozol for 10 min thrice, and ethanol for 5 min thrice. Immunosaver (Nissin EM, Tokyo, Japan) was used for antigen retrieval in accordance with the product data sheet. Slides were blocked with 0.1% sodium azide and 1% FBS containing PBS for 45 min. All sections were treated with the primary antibody solution (1:199 in 0.1% sodium azide and 1% FBS PBS overnight), washed with PBS thrice, and treated with the secondary antibody solution (1:999 in 0.1% sodium azide and 1% FBS containing PBS with Hoechst33342 (10,000 dilution) at room temperature for 45 min). After being washed with PBS thrice, the slides were added with Vectashield. Images were obtained using BZ-X700 (Keyence, Tokyo, Japan) and contrasted or brightness-adjusted in Microsoft PowerPoint (Seattle, WA, USA). Fluorescence intensity levels were assessed using ImageJ (NIH, Bethesda, MD, USA).

### Statistical analyses

Data were expressed as mean ± standard error of the mean (SEM). Significant differences were determined with Dunnett’s test in GraphPad 6.0 (GraphPad Software, Tokyo, Japan) at *P* < 0.05.

## Supplementary Information

Supplementary Information.

## Data Availability

The datasets generated and/or analyzed during the current study are not publicly available but are available from the corresponding author on reasonable request. Humans were not directly involved in the current study and only human cell line was used that was purchased.
