# Supplementary material for: Plant miRNA osa-miR172d-5p suppressed lung fibrosis by targeting Tab1
Source: Sci Rep. 2023 Feb 6;13:2128. doi: 10.1038/s41598-023-29188-6 (PMC9901827; doi:10.1038/s41598-023-29188-6)
Supplement: Supplementary file 1 — Supplementary Information. [file 41598_2023_29188_MOESM1_ESM.pdf]

Plant miRNA osa-miR172d-5p suppressed lung fibrosis by targeting Tab1

Motofumi Kumazoe[1,#], Fumiyo Ogawa[1,#], Ai Hikida[1], Yu Shimada[1], Ren Yoshitomi[1], Ryoya Watanabe[1], Hiroaki Onda[1], Yoshinori Fujimura[1], and Hirofumi Tachibana[1,*]

[1]Division of Applied Biological Chemistry, Department of Bioscience and Biotechnology, Faculty of Agriculture, Kyushu University, Fukuoka 819-0395, Japan

[#]Both authors contributed equally to this work.

Running title: **Antifibrotic effects of Osa-miR172d-5p through Tab1 suppression**

*Corresponding author: Hirofumi Tachibana, 744 Motooka, Nishi-ku, Fukuoka 819-0395, Japan. Tel and Fax: (+81) (92) 802-4749; E-mail: tatibana@agr.kyushu-u.ac.jp

Keywords: plant miR, Fibrosis, Bleomycin, TGFβ, Tab1

SIFig1 The effect of osa-miR172d-5p on the viable cell number of HFL1 cells.

HFL1 cells were treated with indicated miRs (10 nM) for 24 h, 48 h. Viable cells were assessed by ATPlite (n = 4). Data are shown as mean ± SEM. Student's t test.

SIFig2 The effect of osa-miR172d-5p on the expression levels of senescence-related genes and autophagy related genes of HFL1 cells.

(**A-C**) HFL1 cells were treated with indicated miRs (10 nM) for 24 h, 48 h. The expression levels of (**A-B**) senescence-related genes and (**C**) autophagy related genes were assessed by qRT-PCR (n = 4). Data are shown as mean ± SEM. Student's t test. $^{**}P < 0.01$.

SIFig3 The effect of osa-miR172d-5p on the Human Pulmonary Artery Endothelial Cells.

(**A**) Human Pulmonary Artery Endothelial Cells (HPAECs) were treated with indicated miRs (10 nM) for 48 h. Viable cells were assessed by ATPlite (n = 4). (**B-D**) HPAECs were treated with indicated miRs (10 nM) for 48 h. The expression levels of (**B**) TGF related gene, (**C**) senescence-related gene and (**D**) autophagy related gene were assessed by qRT-PCR (n = 4). Data are shown as mean ± SEM. Student's t test.

SIFig4 Osa-miR172d-5p suppressed Col1a upregulation in a bleomycin-induced fibrosis model.

C57BL male mice were treated with bleomycin (1 mg/kg bw intratracheal administration); after 1 week, the bleomycin-treated mice were treated with the negative control miR (3 nmol/mice 5 times every 2 weeks, intraperitoneal injection of AteloGene^{TM}) or miR Osa-miR172d-5p mimic miR (3 nmol/mice five times every 2 weeks, intraperitoneal injection of AteloGene^{TM}). After 3 weeks, the mice were sacrificed, their lung tissues were harvested, and Col1a levels were assessed by western blot analysis (n = 5, 5, 4). Data are shown as mean ± SEM. Dunnett's test. $^{***}P < 0.001$.

SIFig5 Western Blot images

Western Blot images A, B (for Fig1C), C, D (for Fig3A), E and F (for SIFig4A).

SI Table1   Primer List

P21 (CDKN1A)	F	**5'**	TCAAATCGTCCAGCGACCTTC	**3'**
	R	**5'**	CATGCCCTGTCCATAGCCTCTAC	**3'**
IL-1B	F	**5'**	GAAGCTGATGGCCCTAAACA	**3'**
	R	**5'**	AAGCCCTTGCTGTAGTGGTG	**3'**
GLS	F	**5'**	CAGAAGGCACAGACATGGTTGG	**3'**
	R	**5'**	GGCAGAAACCACCATTAGCCAG	**3'**
IL-6	F	**5'**	CCTGAACCTTCCAAAGATGGC	**3'**
	R	**5'**	TTCACCAGGCAAGTCTCCTCA	**3'**
CHOP	F	**5'**	TGTTAAAGATGAGCGGGTGGC	**3'**
	R	**5'**	CTTGAACACTCTCTCCTCAGGTTC	**3'**
ATG9B	F	**5'**	AACTGCTTCGCTCAGTCTGC	**3'**
	R	**5'**	AGCTCAGCTCCTCCGGG	**3'**
ATG16L2	F	**5'**	ACCTGCGTGTCAGCAACAT	**3'**
	R	**5'**	CAGCTTTGGTCCAGTCAGAAC	**3'**
BECN1	F	**5'**	GATGGTGTCTCTCGCAGATTC	**3'**
	R	**5'**	AGTCTTCGGCTGAGGTTCTCC	**3'**
ACTB	F	**5'**	ATGTGGCCGAGGACTTTGATT	**3'**
	R	**5'**	AGTGGGGTGGCTTTTAGGATG	**3'**

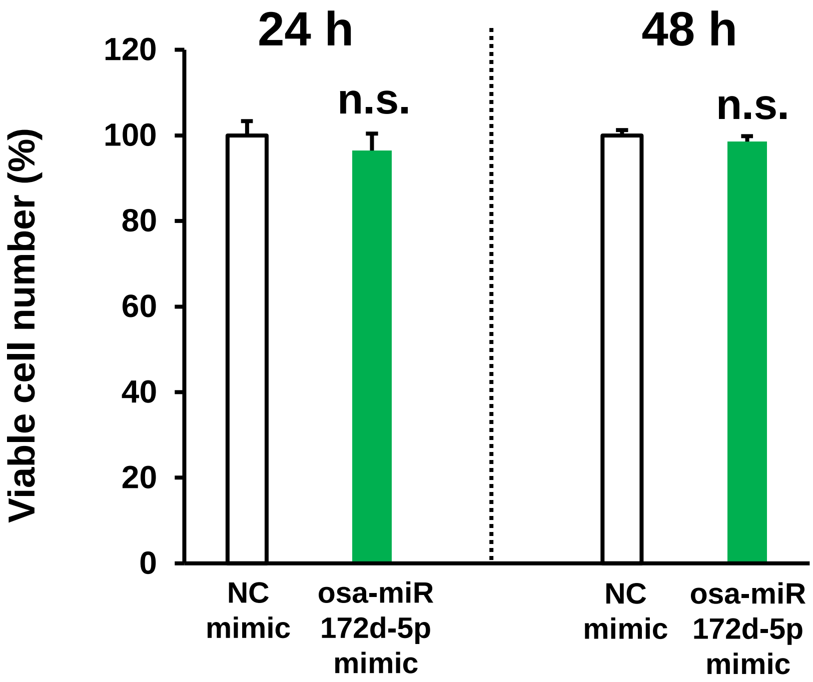
24 h
48 h
Viable cell number (%)
120
100
80
60
40
20
0
n.s.
n.s.
NC mimic
osa-miR 172d-5p mimic
NC mimic
osa-miR 172d-5p mimic

SIFig1

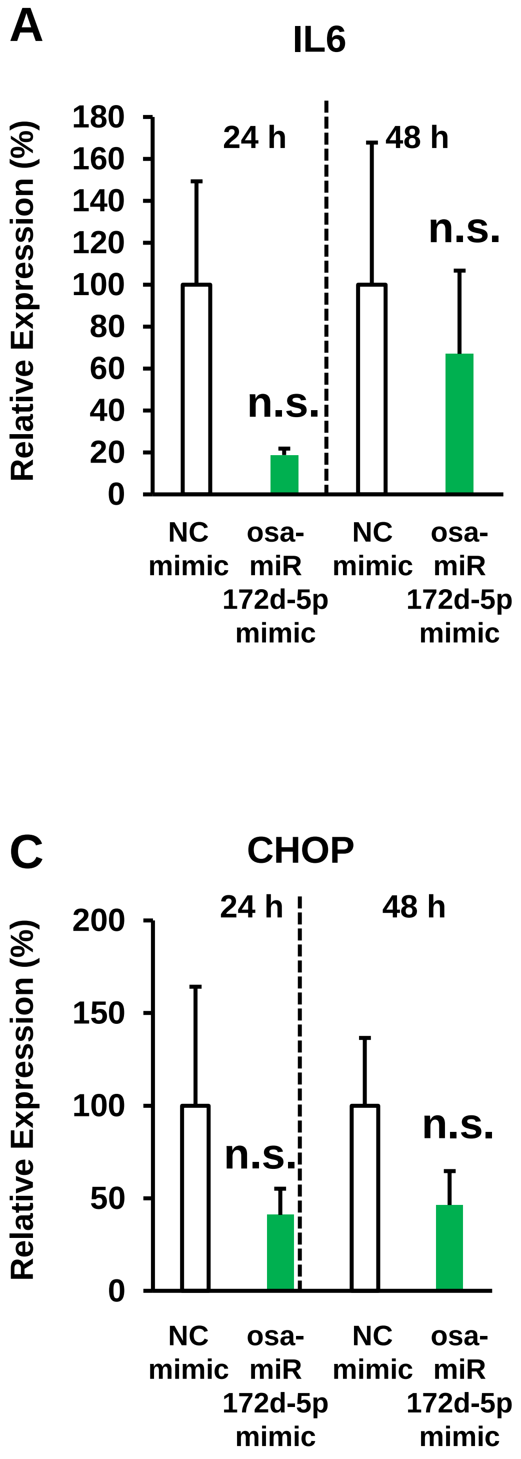
A
IL6
Relative Expression (%)
180
160
140
120
100
80
60
40
20
0
24 h
48 h
n.s.
n.s.
NC mimic
osa-miR 172d-5p mimic
NC mimic
osa-miR 172d-5p mimic
B
P21
Relative Expression (%)
120
100
80
60
40
20
0
24 h
48 h
n.s.
**
NC mimic
osa-miR 172d-5p mimic
NC mimic
osa-miR 172d-5p mimic
C
CHOP
Relative Expression (%)
200
150
100
50
0
24 h
48 h
n.s.
n.s.
NC mimic
osa-miR 172d-5p mimic
NC mimic
osa-miR 172d-5p mimic

SIFig2

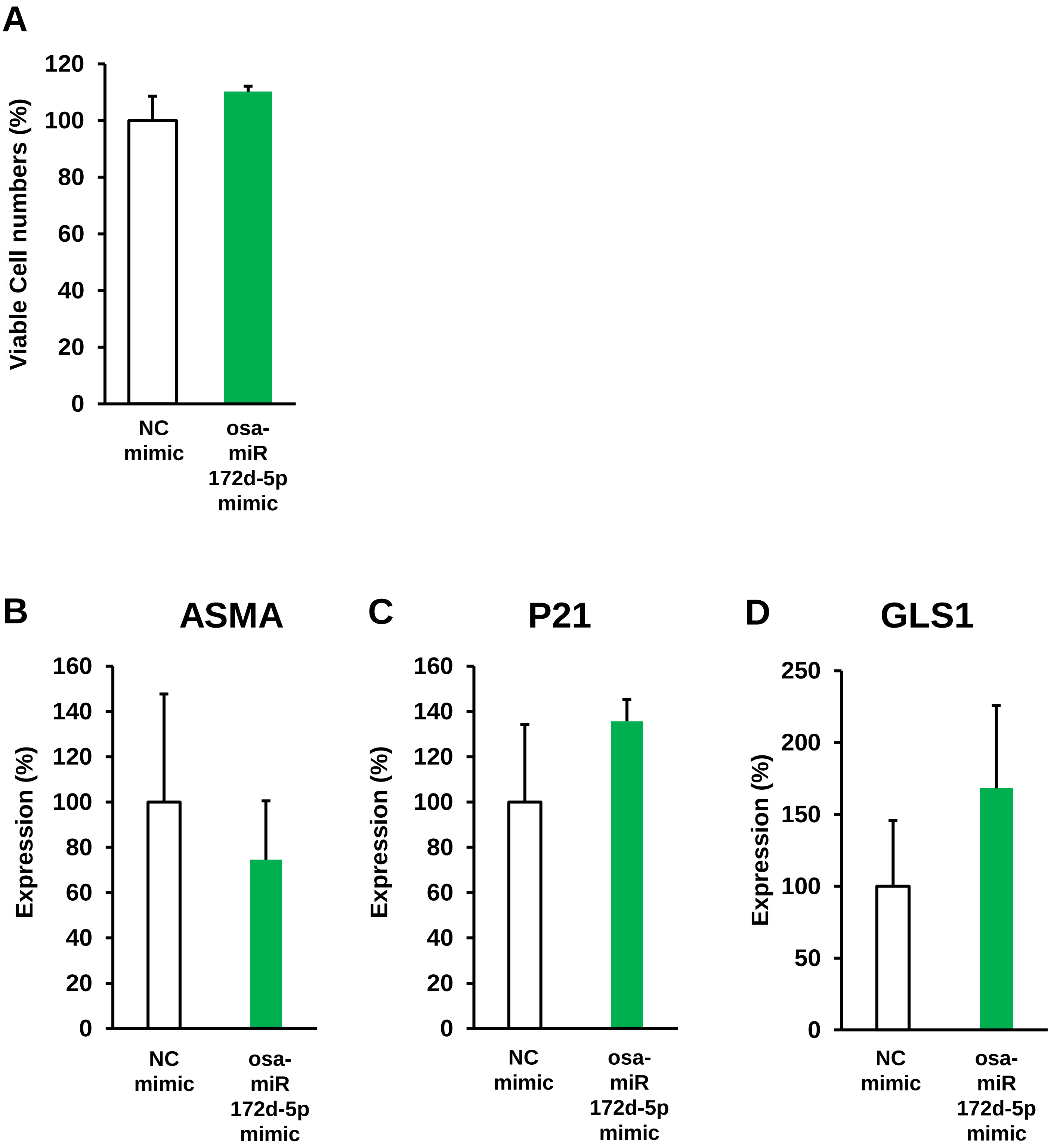

A
Viable Cell numbers (%)
0
20
40
60
80
100
120
NC mimic
osa-miR 172d-5p mimic
B
ASMA
Expression (%)
0
20
40
60
80
100
120
140
160
NC mimic
osa-miR 172d-5p mimic
C
P21
Expression (%)
0
20
40
60
80
100
120
140
160
NC mimic
osa-miR 172d-5p mimic
D
GLS1
Expression (%)
0
50
100
150
200
250
NC mimic
osa-miR 172d-5p mimic

SIFig3

A

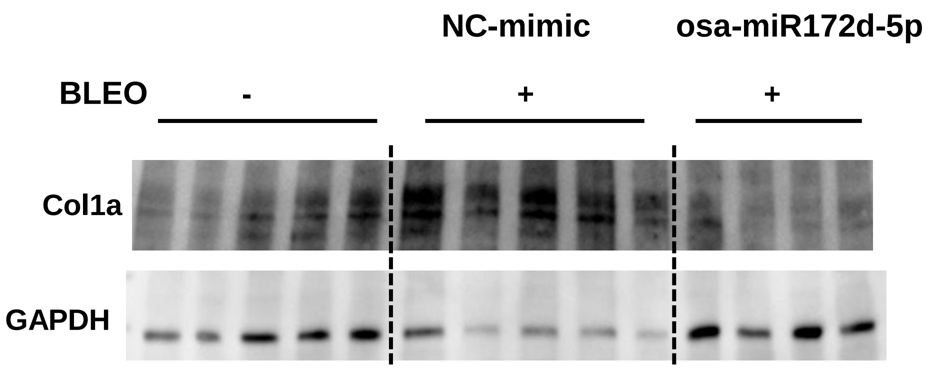
NC-mimic
osa-miR172d-5p
BLEO
-
+
+
Col1a
GAPDH

B

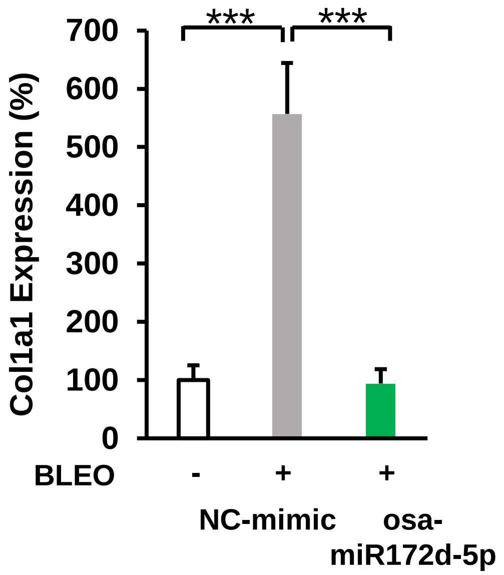
Col1a1 Expression (%)
700
600
500
400
300
200
100
0
***
***
BLEO
-
+
+
NC-mimic
osa-miR172d-5p

SIFig4

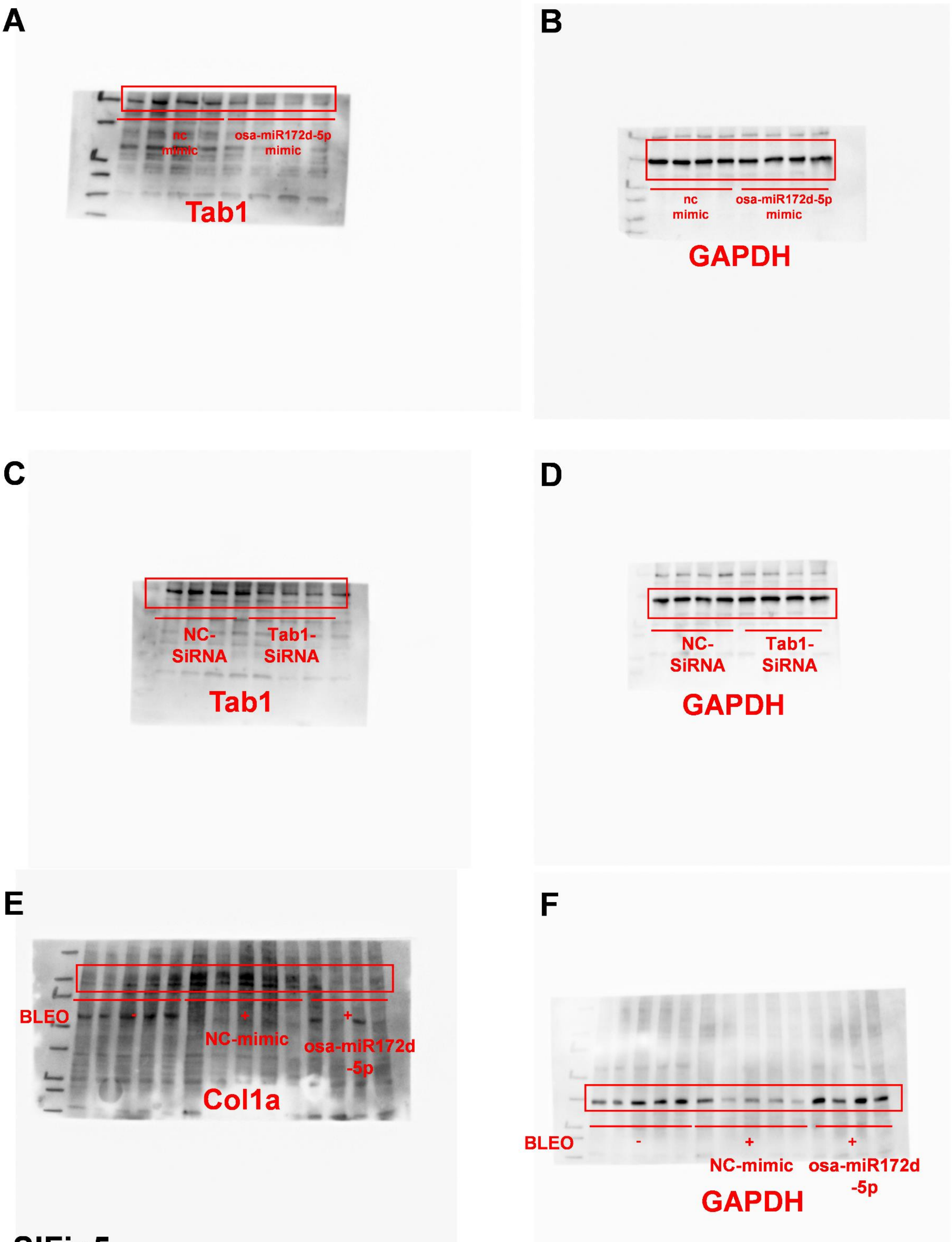
A
nc
mimic
osa-miR172d-5p
mimic
Tab1
B
nc
mimic
osa-miR172d-5p
mimic
GAPDH
C
NC-
SiRNA
Tab1-
SiRNA
Tab1
D
NC-
SiRNA
Tab1-
SiRNA
GAPDH
E
BLEO
-
+
NC-mimic
+
osa-miR172d
-5p
Col1a
F
BLEO
-
+
NC-mimic
+
osa-miR172d
-5p
GAPDH

SIFig5
